# Impact of exposures to Heated Tobacco Products in the media and through social connections on product perceptions and use

**DOI:** 10.18332/tpc/187246

**Published:** 2024-05-10

**Authors:** Lorien C. Abroms, Zongshuan Duan, Yael Bar-Zeev, Yuxian Cui, Yan Wang, Cassidy R. LoParco, Amal Khayat, Hagai Levine, Carla J. Berg

**Affiliations:** 1 Department of Prevention and Community Health, Milken Institute School of Public Health, George Washington University, Washington, DC, United States; 2 Department of Population Health Sciences, School of Public Health, Georgia State University, Atlanta, GA, United States; 3 Braun School of Public Health and Community Medicine, Faculty of Medicine, The Hebrew University of Jerusalem and Hadassah, Jerusalem, Israel; 4 The George Washington University Cancer Center, George Washington University, Washington, DC, United States

**Keywords:** advertising, promotions, media, tobacco, heated tobacco product, IQOS

## Abstract

**INTRODUCTION:**

Little is known about media exposures to heated tobacco products (HTPs). In this study, we examined sources of HTP exposure, including from paid and unpaid media and social connections, in relation to HTP use and use intentions.

**METHODS:**

In the fall of 2021, we conducted a cross-sectional survey among adult online panelists (aged 18–45 years) in the US and Israel, oversampling tobacco users. The current study analyzed data from participants who responded to the question about HTP awareness or use (n=2061). Multivariable linear and logistic regression analyses examined the relationship between sources of HTP exposure, HTP use, and use intentions.

**RESULTS:**

Among those aware of HTPs, both Israelis and Americans reported past-month HTP media exposure via advertisements (58.2% vs 48.0%), non-advertisement sources (49.7% vs 30.7%), and social connections (51.5% vs 33.6%), respectively. Factors associated with HTP awareness (n=677/2061; 32.9%) included media use frequency (AOR=1.13; 95% CI: 1.01–1.28) and social connections using HTPs (AOR=2.45; 95% CI: 1.92–3.15). Among those aware of HTPs, past-month HTP exposure via digital media advertisements (AOR=2.06; 95% CI: 1.09–3.91) and non-advertising promotion via radio, podcast, movie, television or theatre (AOR=2.30; 95% CI: 1.19–4.44) and websites (AOR=2.36; 95% CI: 1.32–4.21) were associated with current HTP use. Exposure to digital media advertisements (β=0.35; 95% CI: 0.07–0.62) and non-advertising promotion via social media (β=0.62; 95% CI: 0.34–0.91) were correlated with higher use intentions. Having social connections using HTPs was correlated with higher use (AOR=2.21; 95% CI: 1.19–4.11) and intentions (β=0.66; 95% CI: 0.42–0.91). No significant differences were found across countries.

**CONCLUSIONS:**

Digital media (e.g. online, social media) were particularly salient correlates of HTP intentions and use. Future studies are needed that further examine media exposures to these products, as well as that examine possible regulations to limit HTP promotion via these channels.

## INTRODUCTION

Tobacco companies are introducing novel tobacco products to consumers by using sophisticated marketing tactics^1^. Heated tobacco products (HTPs) are a relatively new class of tobacco products that use an electronic device to heat a stick of compressed tobacco to produce an inhalable aerosol. HTPs are marketed as a safer alternative to traditional combustible cigarettes^2^, though the science of their safety has been disputed and is still under investigation^3,4^.

The world’s leading HTP is Philip Morris International’s IQOS, which is sold in over 70 countries. IQOS is typically marketed using a mix of print and online advertising, point-of-sale retail marketing, and event marketing^4-6^. IQOS also maintains a company-sponsored social media presence, which includes advertising, event promotions, price promotions, and product use instructions^7^. While IQOS marketing maintains many commonalities across countries, local factors, including those related to historical, cultural, and regulatory differences, likely contribute to some cross-country differences^8^.

In Israel, IQOS is the only HTP on the market. It entered the market in 2016 and is currently sold online at IQOS specialty shops and in retail settings where tobacco products are commonly sold (e.g. gas stations, corner shops)^9^. While IQOS and other HTPs initially experienced minimal marketing restrictions, since March 2019, like other tobacco products in Israel, HTP marketing restrictions are in place, including an advertisement ban (excluding print media), a requirement for plain packaging, and a prohibition on point-of-sale displays (effective January 2020)^10^. Despite restrictions, recent marketing efforts have included a mix of print and online advertising, retail and online sales, event marketing, and a social media presence^10-13^.

In the US, IQOS entered the market in 2019 in the state of Georgia^12^. By 2021, it was sold in Georgia, North Carolina, South Carolina, and Virginia, and was 1 of 2 HTPs available on the US market. At its peak, IQOS marketing efforts included a mix of national print and online advertising, retail sales in 4 states including over 400 kiosks and corner stores, and a social media presence^8,13^. While US distribution was in the process of expanding, on November 29th, 2021, IQOS sales were halted because of a patent dispute with RJR Reynolds, but may re-enter the US market in 2024^6^.

According to the Diffusion of Innovation Theory (DOI)^14^, one key factor determining the rate of adoption of a new product is the communication channels through which information about the product spreads. According to DOI, communication channels, such as television, radio, print advertisements, and social media, play an important role in spreading awareness and adoption of a new product such as IQOS^14^. Interpersonal networks and word of mouth are also considered important, especially for product adoption^14^.

Consistent with DOI, prior research has demonstrated the importance of a person’s social and media environments with regard to adopting the use of tobacco products, including cigarettes, e-cigarettes, and others^15-17^. For example, exposure to tobacco advertisements, including in traditional media such as television and print, as well as newer digital marketing strategies such as social media, increased awareness of tobacco products and influenced their adoption, particularly among vulnerable groups, such as youth and young adults, racial/ethnic minorities, low socioeconomic status (SES) groups, women and gender minorities^18-22^. Additionally, having friends, family members, and peers who use tobacco products increases tobacco product adoption; the number of close friends who are smokers is a well-established risk factor for smoking uptake among youth^18,23-25^. Overall, the media and social environment play an important role in tobacco use.

While the link for the effect of the social and media environment is well established for tobacco products, generally speaking, little is known about more novel products such as HTPs. For HTPs, the existing research regarding factors associated with awareness and use is limited to sociodemographic and tobacco use correlates^26-28^. For example, prior research has identified the correlates of HTP use as other tobacco use (e.g. cigarettes, e-cigarettes), male sex, identifying as a sexual orientation minority^8,11,29-31^, and having friends who use tobacco (e.g. cigarettes and e-cigarettes)^32-35^. No studies to date on HTPs have examined media-related factors associated with their use.

Establishing an evidence base regarding the implications of exposure to HTP-related information via media and social networks is important for understanding how people become aware of these products and perceive them. This is particularly important given that HTPs are frequently marketed as harm-reduction products and were authorized by the US Food and Drug Administration to use ‘reduced exposure’ claims in its US marketing from 2020 until its removal from the market^8^. This study aims to address this gap in the literature by examining among US and Israeli adults: 1) awareness of HTPs; 2) sources of exposure to HTPs (e.g. media including advertisement and non-advertisement sources, and close social connections who use HTPs); and 3) associations of exposure in relation to HTP use, use intentions, and perceived addictiveness and harm.

## METHODS

### Study design and participants

From October to December 2021, online survey data were collected among participants recruited from Ipsos panels in the US and Israel. Eligible participants were citizens of the respective countries, aged 18–45 years, and able to speak English (US), or Hebrew or Arabic (Israel). The target sample size (100/country) and composition were based on power analyses to detect small to medium effects in relation to tobacco use outcomes among key sociodemographic groups (i.e. by sex, racial/ethnic group). Purposive sampling was used to achieve about 40% with tobacco use and representation by sex and racial/ethnic group (in the US: 45% White, 25% Black, 15% Asian, and 15% Hispanic; in Israel: 80% Jewish and 20% Arabic).

In the US, the survey primarily employed KnowledgePanel^®^ (KP), a web panel using probability sampling. Recruitment involved random digit dialing and address-based sampling. KP members received cash incentives (about $5 for a 25-minute survey). Out of 4960 recruited panelists, 2397 (48.3%) passed eligibility screening, and 1095 (45.7%) completed the survey. To reach specific subgroup recruitment goals, Ipsos recruited a convenience sample of US adults reporting Asian race and tobacco use (via banner advertisements, web pages, and emails). Among 353 screened individuals, 33 (9.3%) were eligible and completed the survey. In Israel, an opt-in sample was used, mirroring the US approach. Out of 2970 individuals who completed eligibility screening and were eligible, 1094 (36.8%) completed the survey. The final sample included 2222 participants (US, n=1128; Israel, n=1094). The current study analyzed data from participants who provided a response to the question about HTP awareness or use (N=2061; US, n=1049; Israel, n=1012).

Additional details about the study have been reported previously^12^. This study was approved by the Institutional Review Boards of George Washington University, USA (NCR213416) and the Hebrew University, Israel (27062021) and complies with the Strengthening the Reporting of Observational Studies in Epidemiology (STROBE) reporting guidelines for cross-sectional studies.

### Measures

*Dependent variables*

This study’s 5 outcome variables included HTP awareness, current use, use intentions, perceived harm, and perceived addictiveness. For HTP awareness, we used the following procedures and items. At the beginning of the survey, we displayed images of IQOS devices, device chargers, and heatsticks, and briefly described HTPs and IQOS: ‘The following questions are aimed at learning more about your perceptions of heated tobacco products. These products heat tobacco but do not actually burn it … One common brand is IQOS (pictured below). IQOS is an electronic device and has 3 main parts: 1) the device charger; 2) the IQOS device (or heatstick ‘holder’) into which heatsticks are inserted and heated; and 3) the heatsticks that contain tobacco and are inserted into the IQOS device to be heated’. Then, we assessed prior awareness of HTPs among all participants by asking: ‘Before beginning this survey, had you heard of heated tobacco products, like IQOS, which heat sticks of tobacco instead of burning it?’ (yes, no, don’t know). Those who reported ‘yes’ to awareness of HTPs on this item were coded as aware of HTPs.

To assess current HTP use, all participants were asked: ‘In your lifetime, have you ever used heated tobacco products, which are devices that heat actual tobacco but do not burn tobacco, such as IQOS?’ (yes vs no). Among those reporting yes to HTP ever use, current HTP use was assessed by asking: ‘In the past 30 days, how many days have you used heated tobacco products?’ (0–30, recategorized as yes [≥1 day] or no [0 days]). HTP use intentions were assessed by asking: ‘How likely are you to try or continue to use heated tobacco products in the next year?’ (1=not at all, to 7=extremely). Finally, perceived addictiveness and harm of HTPs were assessed by asking: ‘How (addictive/harmful) do you think heated tobacco products (such as IQOS) are?’ (1=not at all, to 7=extremely).

*Independent variables*

Only those reporting ‘yes’ to awareness of HTPs (n=677) were asked the following series of additional questions regarding their recall of any exposure to HTPs in their social connections or via advertisements and non-advertisements. Close social connections using HTPs within participants’ social environments were measured by asking: ‘How many of your closest connections (including your partner, friends, relatives, co-workers, and others) use a heated tobacco product like IQOS or Eclipse?’ (1=none, to 7=all, recategorized as none vs any). Advertisement exposure via 11 media channels was assessed by asking: ‘In the last 30 days, have you noticed heated tobacco product (like IQOS) advertisements in any of the following places: 1) websites; 2) social media; 3) inside tobacco stores; 4) outside tobacco stores; 5) vape shops; 6) television; 7) radio; 8) posters, billboards, etc.; 9) newspapers or magazines; 10) direct mail; and 11) email’ (yes, no). These 11 media channels were categorized as 1) digital media (i.e. websites, social media, direct mail, email); 2) traditional media (i.e. television, radio, newspapers or magazines, posters, billboards, etc.); and 3) retail setting (i.e. inside or outside tobacco shops, or vape shops). To categorize the extent of exposure, we also created a sum score for advertisement exposure across media platforms (range: 0–11) and a variable of whether there was any exposure across platforms in the past 30 days (yes/no). Non-advertisement exposure via four types of media channels was assessed by asking: ‘Outside of advertisements, in the last 30 days, have you noticed heated tobacco products (like IQOS) being referenced, used, or portrayed in any of the following places: 1) movies, television, or theater; 2) radio; 3) websites; and 4) social media’ (yes, no). Any exposure to HTP advertisements and non-advertisements (yes vs no) were coded, respectively. To categorize the extent of exposure, we created a sum score for non-advertisement exposure across media platforms (range: 0–4) and a variable of whether there was any exposure across platforms in the past 30 days (yes/no). To characterize first exposure (descriptive measures only), we asked: ‘How did you first learn about heated tobacco products, such as IQOS: saw advertisements, saw the product at a store, friends/family/colleagues, saw their use on TV, movies, etc., saw posts on social media (not advertisements), don’t know/remember’.

*Covariates*

Covariates included in the final models include country (Israel, United States), age, sex (male, female), sexual orientation (heterosexual, not heterosexual), education level (< college degree, ≥ college degree), relationship status (married/living with partner, other), children in the home (yes, no), and current use (yes, no) of cigarettes, e-cigarettes, and other tobacco products (i.e. hookah, cigar, pipe, smokeless tobacco). Frequency of media use was also included and assessed by asking: ‘How often, if at all, do you use the following media sources: newspapers, magazines; television; radio, news podcasts; news websites; and social media’ (1=never, to 6=almost constantly).

*Descriptive variable*

An additional variable, race/ethnicity (US: White, Black, Hispanic, Asian or other; Israel: Jewish, Arabic), was examined for descriptive purposes only. Race/ethnicity was not included in the final models as there was little overlap in racial/ethnic categories between the racial/ethnic categories in the US (e.g. White) and Israel (e.g. Jewish), and because in country-specific models, no racial/ethnic associations were found.

### Data analysis

All data management and analyses were conducted using Stata 15.1 (StataCorp LLC, College Station, TX, USA). Descriptive and bivariate analyses were conducted to characterize participants overall and by country (chi-squared tests for categorical variables and t-tests or ANOVAs for continuous variables).

Among all participants in our analytic sample (N=2061), multivariable logistic regression was conducted to assess associations between participant characteristics and HTP awareness. Among the sub-set of participants who reported ‘yes’ to HTP awareness (n=677), we assessed advertisement exposure, non-advertisement exposure, and close connections using HTPs in relation to: 1) past-month HTP use via multivariable logistic regression; and 2) use intentions, perceived addictiveness, and perceived harm via multivariable linear regression analyses controlling for covariates (i.e. country, age, sex, sexual orientation, education level, relationship status, children in the home, and current use of cigarettes, e-cigarettes, and other tobacco products.). Multiplicative interaction terms between country and primary exposures were tested for all models. All statistical tests were 2-tailed with a significance level set at α=0.05.

## RESULTS

As shown in Table 1, 24.3% of participants were aged 18–25 years, 37.1% were 26–35 years, and 38.7% were 36–45 years. Half of the participants were female (50.0%) and had at least a college degree (50.1%). Over half were married (54.2%) and lived with children aged <18 years (51.1%). The most common type of tobacco currently used was cigarettes (31.6%) and e-cigarettes (20.3%). Overall, 32.9% reported HTP awareness (Israel 42.8%, US 23.3%), and 8.0% currently used HTPs (Israel 12.8%, US 3.2%).

**Table 1:** HTP awareness by media use, tobacco use, and demographic factors, among adults in US and Israel (N=2061)

	*Total*	*Overall (N=2061)*			*US (N=1049)*			*Israel (N=1012)*		
		*Yes*	*No*		*Yes*	*No*		*Yes*	*No*	
	*n (%)*	*n (%)*	*n (%)*	*p*	*n (%)*	*n (%)*	*p*	*n (%)*	*n (%)*	p*
**Total**	2061 (100)	677 (32.9)	1384 (67.2)		244 (23.3)	805 (76.7)		433 (42.8)	579 (57.2)	
**Demographics**										
**Age** (years)										
18–25	500 (24.3)	152 (22.5)	348 (25.1)	0.280	29 (11.9)	117 (14.5)	0.550	123 (28.4)	231 (39.9)	**0.001**
26–35	764 (37.1)	265 (39.1)	499 (36.1)		93 (38.1)	306 (38.0)		172 (39.7)	193 (33.3)	
36–45	797 (38.7)	260 (38.4)	537 (38.8)		122 (50.0)	382 (47.5)		138 (31.9)	155 (26.8)	
**Female**	1030 (50.0)	269 (39.7)	761 (55)	**<0.001**	103 (42.2)	418 (51.9)	**0.008**	166 (38.3)	343 (59.2)	**<0.001**
**Heterosexual**	1759 (85.4)	572 (84.5)	1187 (85.8)	0.420	208 (85.3)	711 (88.4)	0.184	364 (84.1)	476 (82.2)	0.437
**Race/Ethnicity**										
White, Non-Hispanic (US)	462 (44.0)	112 (45.9)	350 (43.5)	0.772	112 (45.9)	350 (43.5)	0.772	-	-	
Black, Non-Hispanic (US)	254 (24.2)	57 (23.4)	197 (24.5)		57 (23.4)	197 (24.5)		-	-	
Other, Non-Hispanic (US)	168 (16.0)	41 (16.8)	127 (15.8)		41 (16.8)	127 (15.8)		-	-	
Hispanic (US)	165 (15.7)	34 (13.9)	131 (16.3)		34 (13.9)	131 (16.3)		-	-	
Arabic (Israel)	126 (12.5)	62 (14.3)	64 (11.1)	0.120	-	-		62 (14.3)	64 (11.1)	0.120
Jewish (Israel)	886 (87.6)	371 (85.7)	515 (89.0)		-	-		371 (85.7)	515 (89.0)	
**Education level^a^ < College degree**	1032 (50.1)	360 (53.2)	672 (48.6)	**0.049**	107 (43.9)	349 (43.4)	0.891	253 (58.4)	323 (55.8)	0.401
**Married/living with partner**	1116 (54.2)	394 (58.2)	722 (52.2)	**0.010**	138 (56.6)	429 (53.3)	0.370	256 (59.1)	293 (50.6)	**0.007**
**Living with children aged <18 years**	1053 (51.1)	384 (56.7)	669 (48.3)	**<0.001**	122 (50.0)	377 (46.8)	0.385	262 (60.5)	292 (50.4)	**0.001**
**Media use frequency**, mean (SD)	3.44 (1.00)	3.75 (1.01)	3.28 (0.96)	**<0.001**	3.48 (1.04)	3.06 (0.92)	**<0.001**	3.91 (0.95)	3.60 (0.93)	**<0.001**
Newspapers, magazines	2.42 (1.42)	2.92 (1.60)	2.18 (1.26)	**<0.001**	2.56 (1.53)	1.91 (1.08)	**<0.001**	3.13 (1.61)	2.55 (1.40)	**<0.001**
Television	3.74 (1.62)	3.97 (1.58)	3.63 (1.63)	**<0.001**	3.86 (1.60)	3.41 (1.56)	**<0.001**	4.04 (1.57)	3.93 (1.68)	0.311
Radio, news podcasts	3.15 (1.52)	3.48 (1.52)	2.98 (1.49)	**<0.001**	3.37 (1.54)	2.91 (1.45)	**<0.001**	3.54 (1.51)	3.08 (1.54)	**<0.001**
News websites	3.60 (1.53)	3.92 (1.48)	3.45 (1.53)	**<0.001**	3.56 (1.50)	3.23 (1.49)	**0.001**	4.12 (1.43)	3.76 (1.54)	**<0.001**
Social media	4.30 (1.64)	4.45 (1.55)	4.22 (1.68)	**<0.001**	4.02 (1.64)	3.84 (1.66)	0.137	4.69 (1.44)	4.75 (1.58)	0.596
**HTP and other tobacco use**										
**HTP use**										
Never	1770 (86.3)	505 (75.2)	1265 (91.8)	**<0.001**	204 (85.0)	773 (96.6)	**<0.001**	301 (69.7)	492 (85.1)	**<0.001**
Former	116 (5.7)	61 (9.1)	55 (4.0)		11 (4.6)	18 (2.3)		50 (11.6)	37 (6.4)	
Current	164 (8.0)	106 (15.8)	58 (4.2)		25 (10.4)	9 (1.1)		81 (18.8)	49 (8.5)	
**Current cigarette use**	643 (31.6)	330 (49.7)	313 (22.9)	**<0.001**	91 (39.1)	148 (18.7)	**<0.001**	239 (55.5)	165 (28.5)	**<0.001**
**Current e-cigarette use**	413 (20.3)	238 (35.7)	175 (12.8)	**<0.001**	65 (27.5)	92 (11.7)	**<0.001**	173 (40.1)	83 (14.4)	**<0.001**
**Current other tobacco use^b^**	452 (22.3)	228 (34.4)	224 (16.4)	**<0.001**	69 (29.9)	90 (11.5)	**<0.001**	159 (36.8)	134 (23.2)	**<0.001**

a Income and employment also not significantly associated, so not included.

b Other tobacco: hookah, cigar, pipe, smokeless tobacco.

* Boldface indicates p<0.05.

**Figure 1:**
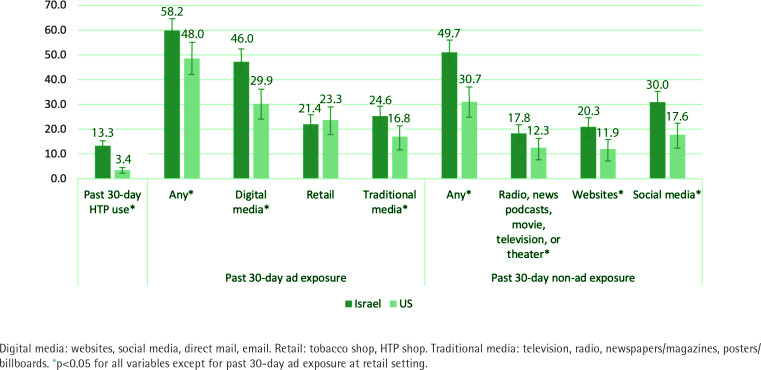
Past 30-day HTP use, exposure to advertisements and non-advertising promotion by country (N=677)

As shown in Table 2, among the subset who reported being aware of HTPs (n=677), the source of first exposure was most commonly friends, family, or colleagues (33.2%), followed by advertisements (23.7%), stores (14.3%), social media (non-advertisements) (9.2%), and TV (non-advertisements) (3.3%). The pattern was different across countries: those in Israel were more likely to report first exposure from friends or family, and those in the US were more likely to say that they don’t know/remember where they first learned about HTPs (p<0.001). Additionally, 47.0% of those who were aware of HTPs reported having at least one close social connection using HTPs, with 51.5% in Israel and 33.6% in the US (p<0.001). Regarding past-month HTP exposure, 55.8% reported any past-month advertisement exposure, with 48.0% in the US and 58.8% in Israel (p<0.01). In terms of past-month advertisements, 41.2% reported digital media exposure (i.e. websites, social media, direct mail, email), 22.6% at retail settings (i.e. inside or outside tobacco shop, vape shop), and 22.3% via traditional media (i.e. television, radio, newspapers/magazines, posters/billboards). Regarding non-advertisements, 43.9% reported any past-month non-advertisement exposure (US 30.7%, Israel 49.7%, p<0.001), including 26.2% via social media, 17.7% via websites, and 16.2% via radio and news podcasts, movie, television, or theater (Table 2 and Figure 1).

**Table 2:** Sources of past-month HTP exposure and first exposure among adults who were aware of HTPs in US and Israel (N=677)

	*Overall (N=677) n (%)*	*US (N=244) n (%)*	*Israel (N=443) n (%)*	p*
**First learned about HTPs**				
Friends or family or colleagues	213 (33.2)	49 (20.1)	164 (37.0)	**<0.001**
Saw advertisements	152 (23.7)	52 (21.3)	100 (22.6)	
Saw product at store	92 (14.3)	35 (14.3)	57 (12.9)	
Saw posts on social media (not ads)	59 (9.2)	18 (7.3)	41 (9.3)	
Saw their use on TV, movies, etc.	21 (3.3)	8 (3.3)	13 (2.9)	
Don’t know/remember	105 (16.4)	64 (26.2)	41 (9.3)	
**Close social connections using HTPs**	310 (47.0)	82 (33.6)	228 (51.5)	**<0.001**
**Past-month HTP advertising exposure**	375 (55.8)	117 (48.0)	258 (58.2)	**0.005**
Digital media	277 (41.2)	73 (29.9)	204 (46.0)	**<0.001**
Retail	152 (22.6)	57 (23.3)	95 (21.4)	0.632
Traditional media	150 (22.3)	41 (16.8)	109 (24.6)	**0.013**
Number of media platforms (0–11), mean (SD)	1.16 (1.52)	1.29 (1.55)	0.93 (1.43)	**0.004**
**Past-month HTP non-advertising exposure**	295 (43.9)	75 (30.7)	220 (49.7)	**<0.001**
Radio, news podcasts, movie, TV, theater	109 (16.2)	30 (12.3)	79 (17.8)	**0.047**
Website	119 (17.7)	29 (11.9)	90 (20.3)	**0.004**
Social media	176 (26.2)	43 (17.6)	133 (30.0)	**<0.001**
Number of media platforms (0–4), mean (SD)	0.64 (0.89)	0.74 (0.89)	0.45 (0.84)	**<0.001**
**Intention to use HTPs**, mean (SD)	2.30 (1.89)	1.81 (1.56)	2.55 (1.99)	**<0.001**
**Perceived addictiveness of HTPs**, mean (SD)	5.00 (1.99)	5.10 (2.07)	4.94 (1.95)	0.345
**Perceived harm of HTPs**, mean (SD)	5.25 (1.83)	5.31 (1.75)	5.22 (1.88)	0.576

* Boldface indicates p<0.05.

Displayed in Table 3, multivariable logistic regression indicated that, controlling for other variables in the model, factors associated with HTP awareness included media use frequency (AOR=1.13; 95% CI: 1.01–1.28), past-month use of cigarettes (AOR=1.72; 95% CI: 1.34–2.21), e-cigarettes (AOR=2.02; 95% CI: 1.51–2.68), having close connections using HTPs (AOR=2.45; 95% CI: 1.92–3.15), being from Israel (vs US) (AOR=2.00, 95% CI: 1.58–2.56), and being male (AOR=1.82; 95% CI: 1.45–2.27).

**Table 3:** Adjusted associations of HTP awareness among adults in US and Israel, overall and by country

	*Overall (N=1883)*		*US (N=930)*		*Israel (N=953)*	
	*AOR*	*95% CI*	*AOR*	*95% CI*	*AOR*	*95 % CI*
**Close connections using HTPs**	**2.45**	**1.92–3.15**	**3.26**	**2.18–4.88**	**2.10**	**1.53–2.88**
**Media use frequency**	**1.13**	**1.01–1.28**	**1.28**	**1.06–1.53**	1.01	0.86–1.19
**Current tobacco use status** (Ref: No)						
Cigarette	**1.72**	**1.34–2.21**	**1.58**	**1.06–2.36**	**1.82**	**1.31–2.53**
E-cigarette	**2.02**	**1.51–2.68**	1.57	**0.99–2.48**	**2.47**	**1.69–3.60**
Other tobacco products*	1.08	0.82–1.43	**1.66**	**1.05–2.64**	0.83	0.58–1.19
**Demographics**						
Israel (Ref: US)	**2.00**	**1.58–2.56**	-	-	-	-
Age (years) (Ref: 36–45)						
18–25	**0.62**	**0.45–0.85**	0.66	0.37–1.19	**0.64**	**0.43–0.96**
26–35	1.00	0.78–1.29	0.90	0.63–1.31	1.09	0.77–1.56
Male (Ref: Female)	**1.82**	**1.45–2.27**	**1.51**	**1.07–2.12**	**2.12**	**1.61–2.86**
Sexual minoritized (Ref: Heterosexual)	1.09	0.80–1.48	1.28	0.76–2.15	0.99	0.67–1.46
Less than college (Ref: College or higher)	1.00	0.80–1.26	1.13	0.79–1.60	0.90	0.67–1.23
Married/living with partner (Ref: No)	0.94	0.74–1.21	1.00	0.68–1.47	0.91	0.65–1.27
Living with children aged <18 years (Ref: No)	**1.40**	**1.11–1.77**	1.22	0.85–1.76	**1.56**	**1.14–2.14**

AOR: adjusted odds ratio. Analyses control for the following variables: country, age, sex, sexual orientation, educational level, relationship status, children in the home, and current use of cigarettes, e-cigarettes, and other tobacco products.

* Other tobacco: hookah, cigar, pipe, smokeless tobacco. Differences in N relative to Table 1 due to selection of missing predictor variables. Boldface indicates p<0.05.

Multivariable logistic regression (Table 4) showed that controlling for other variables in the model, factors associated with current HTP use included past-month HTP advertisement exposure via digital media (AOR=2.06; 95% CI: 1.09–3.91) and non-advertising promotion exposure via radio, podcast, movie, television or theatre (AOR=2.30; 95% CI: 1.19–4.44) and websites (AOR=2.36; 95% CI: 1.32–4.21), and having close connections using HTPs (AOR=2.21; 95% CI: 1.19–4.11). Regarding next-year HTP use intentions and risk perceptions (i.e. addictiveness and harm), past-month advertisement exposure via digital media (β=0.35; 95% CI: 0.07–0.62), non-advertising promotion exposure via social media (β=0.62; 95% CI: 0.34–0.91), having close connections using HTPs (β=0.66; 95% CI: 0.42–0.91) were associated with higher use intentions (Table 4). Past month advertisement exposure via traditional media was associated with lower perceived harm (β= -0.39; 95% CI: -0.76 – -0.03). No other media exposure nor close connection variables were independently associated with perceived harm. Further, neither the media nor close connection variables were significantly associated with perceived addictiveness. No significant interactions were found between media use and close connection variables and country (US vs Israel) in any of the models, indicating the relationships were consistent across the countries.

**Table 4:** Adjusted associations of exposure to HTP in media among participants who reported awareness of HTPs

	*Current HTP use (N=634)*		*Intention to use HTPs (N=619)*		*Perceived addictiveness of HTPs (N=620)*		*Perceived harm of HTPs (N=620)*	
	*AOR*	*95% CI*	*β*	*95% CI*	*β*	*95% CI*	*β*	*95 % CI*
**HTP ad exposure in the past 30 days**								
Digital media (Ref: No)	**2.06**	**1.09–3.91**	**0.35**	**0.07–0.62**	-0.07	-0.45–0.30	-0.09	-0.43–0.26
Retail (Ref: No)	0.98	0.53–1.79	-0.17	-0.45–0.10	0	-0.38–0.37	0	-0.34–0.35
Traditional media (Ref: No)	0.85	0.46–1.59	0.19	-0.10–0.48	-0.28	-0.68–0.12	**-0.39**	**-0.76 – -0.03**
**HTP non-ad exposure in the past 30 days**								
Radio, news podcasts, movie, TV, or theater (Ref: No)	**2.30**	**1.19–4.44**	0.04	-0.29–0.38	-0.12	-0.58–0.34	-0.12	-0.54–0.30
Websites (Ref: No)	**2.36**	**1.32–4.21**	-0.26	-0.58–0.06	-0.40	-0.84–0.03	-0.29	-0.69–0.10
Social media (Ref: No)	0.98	0.55–1.77	**0.62**	**0.34–0.91**	-0.33	-0.72–0.06	-0.24	-0.60–0.11
**Connections using HTPs** (Ref: No)	**2.21**	**1.19–4.11**	**0.66**	**0.42–0.91**	0.27	-0.06–0.61	0.03	-0.28–0.33
**Current tobacco use status**								
HTP (Ref: No)	-	-	**0.86**	**0.52–1.20**	**-0.72**	**-1.18 – -0.25**	**-0.89**	**-1.32 – -0.46**
Cigarette (Ref: No)	**2.64**	**1.37–5.11**	**0.84**	**0.59–1.09**	0.27	-0.07–0.61	0.14	-0.18–0.45
E-cigarette (Ref: No)	**2.69**	**1.48–4.88**	**0.85**	**0.58–1.12**	-0.11	-0.48–0.26	-0.17	-0.51–0.17
Other tobacco products* (Ref: No)	**2.85**	**1.63–4.99**	0.19	-0.08–0.45	-0.16	-0.52–0.20	-0.06	-0.38–0.27
Demographics								
US (Ref: Israel)	0.86	0.46–1.63	**-0.26**	**-0.50 – -0.01**	0.06	-0.28–0.40	-0.08	-0.39–0.23
Age (years) (Ref: 36–45)								
18–25	0.63	0.29–1.35	-0.27	-0.60–0.06	**-0.55**	**-0.99 – -0.10**	-0.27	-0.68–0.13
26–35	0.87	0.47–1.62	-0.21	-0.47–0.04	0.32	-0.03–0.67	0.06	-0.27–0.38
Female (Ref: Male)	1.14	0.66–1.97	**0.33**	**0.10–0.56**	0.17	-0.14–0.48	**0.37**	**0.09–0.65**
Sexual minoritized (Ref: Heterosexual)	0.82	0.38–1.74	-0.03	-0.34–0.28	-0.17	-0.59–0.26	**-0.60**	**-0.99 – -0.21**
Less than college (Ref: College or higher)	**0.58**	**0.33–1.00**	0.17	-0.06–0.40	**-0.46**	**-0.77 – -0.15**	**-0.44**	**-0.72 – -0.15**
Married/living with partner (Ref: No)	0.67	0.36–1.25	0.01	-0.25–0.27	-0.07	-0.42–0.28	-0.17	-0.49–0.16
Living with children aged <18 years (Ref: No)	0.89	0.49–1.62	0.09	-0.15–0.34	0.19	-0.15–0.52	0.16	-0.15–0.47

AOR: adjusted odds ratio. Analyses control for the following variables: country, age, sex, sexual orientation, educational level, relationship status, children in the home, and current use of cigarettes, e-cigarettes, and other tobacco products.

* Other tobacco: hookah, cigar, pipe, smokeless tobacco. Differences in N relative to Table 2 due to selection of missing predictor variables. Boldface indicates p<0.05.

Regarding tobacco use correlates, current HTP use was independently associated with higher use intentions (β=0.86; 95% CI: 0.52–1.20) and lower perceived harm (β= -0.89; 95% CI: -1.32 – -0.46) and addictiveness (β= -0.72; 95% CI: -1.18 – -0.25). Currently using cigarettes or e-cigarettes

were associated with current HTP use (AOR=2.64; 95% CI: 1.37–5.11 and AOR=2.69, 95% CI: 1.48–4.88, respectively) and higher HTP use intentions (β=0.84; 95% CI: 0.59–1.09 and β=0.85; 95% CI: 0.58–1.12, respectively). No significant interactions were found between media use and close connection variables and country (US vs Israel) in any of the models, indicating the relationships were consistent across the countries. No significant interactions were found between tobacco use variables and country (US vs Israel) in any of the models, indicating the relationships were consistent across the countries.

## DISCUSSION

In this sample of US and Israeli adults, awareness of HTPs was moderately high and varied by country, with more awareness in Israel compared with the US. Among people aware of HTPs, those in Israel and the US reported past-month HTP media exposure via advertisements (58.2% vs 48.0%) and other non-advertisement sources (49.7% vs 30.7%) and through their close social connections who used HTPs (51.5% vs 33.6%), respectively. Factors associated with awareness of HTPs included media use frequency, past-month use of cigarettes and e-cigarettes, being male, and having close connections using HTPs. Reports of past-month HTP exposures via digital media advertisements and non-advertising promotions via radio, podcast, movie, television, or theatre and websites, more than doubled the odds of current HTP use. Exposure to digital media advertisements and non-advertising promotions via social media was positively associated with higher use intentions. Exposure to traditional media advertisements was associated with decreased perceived harm of HTPs. Finally, having close connections using HTPs was also associated with an increased likelihood of use and use intentions.

The higher level of media and social exposure to HTPs in Israel compared with the US was expected in light of the differences in market entry and regulatory contexts between countries. IQOS entered the Israeli market three years prior to the US market and quickly reached national distribution. Despite changes in regulations during its market entry (including the elimination of point-of-sale marketing, a partial advertisement ban in 2019, and a change to plain packaging and display ban in 2020), IQOS was able to maintain a national print and online presence in Israel^10^. In the US, IQOS was comparatively new to the market and did not have national distribution. It was only sold in 4 states at the time of the US survey, though there was a national media campaign in place^13^.

Despite these differences across countries in exposure, the relationship observed between exposure to HTPs in the media and from close connections and HTP perceptions and use was similar across countries. In both countries, advertisements seen via digital media and non-advertisements seen on a variety of platforms (i.e. websites, social media, radio, podcasts, movies, TV, or theater) were associated with use and/or intentions to use. Additionally, past 30-day exposure to advertisements via traditional media (e.g. print advertisements in newspapers) was associated with reduced perceptions of the harms of HTPs. Finally, in both countries, having a close connection with those who used HTPs was associated with HTP use and future use intentions. The existence of this similar relationship is consistent with expectations from the Diffusion of Innovation Theory^14^ and implies that similar strategies might be used across countries to reduce HTP exposure and prevent HTP use.

Our study also examined the relative importance of different exposures. From our analysis of paid advertising, digital advertisements had clear effects on intentions to use and use, while retail advertising (e.g. advertisements in stores) and traditional media (e.g. print advertisements) had no significant association. While both print and digital IQOS advertisements were being used at the time of the survey in both countries, this finding implies that digital advertisements may have had greater effects on product use. As such, digital advertisements are worthy of independent consideration in developing product regulations, including bans on digital advertising for novel tobacco products like HTPs. Unlike prior studies of e-cigarettes, which have found effects of unpaid social media exposure on use^24,36^, we did not find an effect of unpaid social media exposure on use, but we did find a positive correlation with intentions to use. As this is one of the first studies to examine media exposure for HTPs, additional studies are warranted.

### Strengths and limitations

The strengths of this study are that with detailed measures of media exposure, including from paid and unpaid sources. The present study is the first, to our knowledge, to examine media related factors related to HTP perceptions and use. Additionally, it was conducted using samples from two countries, making international comparisons possible and inferences more generalizable.

Limitations of this study include the fact that the data are cross-sectional, making causal inference impossible. Future studies are needed to longitudinally examine the effects of media exposure on HTP product uptake. Additionally, the generalizability of our study findings is limited by low participation rates in the survey. Further, findings may be weighted more toward tobacco users given the sampling strategies used, whereby we oversampled those with tobacco use. Thus, awareness of HTPs and exposure to HTPs in the media are likely lower in the general population compared with our sample. Another factor that may have limited awareness is that in the US, IQOS, the leading HTP, was removed from the market at the end of the US survey period in 2021. This may have led to a lower level of exposure to HTPs in our US sample than may have been observed otherwise. Finally, the data are limited by those factors inherent to self-reported surveys and recall of exposures. Participants may have misclassified their exposures to HTP, especially for exposures on social media, where it can be hard to differentiate paid advertising from user-generated content. Some inconsistent survey responses were observed on self-reported awareness, whereby some participants reported being unaware of HTPs but later reported that they used HTPs. These limitations may be inherent to all kinds of survey research assessing awareness. In the case of this study, it meant that fewer participants were asked about the source of HTP media exposure, possibly additionally limiting the generalizability of our sample.

## CONCLUSIONS

This study is notable as one of the first studies to examine a wide array of media and social exposures of HTPs and analyze their association with HTP beliefs, intentions, and use. It establishes that for HTPs, both advertisement and non-advertisement exposures in the media environment, as well as having a close connection using HTPs, are important factors associated with HTP use and/or perceptions. These findings imply that, like for other tobacco products, HTP media influences are salient. Thus, media channels may require surveillance and regulation to limit exposures and subsequent use among vulnerable groups such as youth.

## Data Availability

The data supporting this research are available from the authors upon reasonable request.
